# How Much of Hazardous Blue Light is Transmitted By Spectacle Lenses?

**DOI:** 10.18502/jovr.v15i3.7465

**Published:** 2020-07-29

**Authors:** Saeed Rahmani, Mohammadreza Nazari, Alireza Akbarzadeh Baghban, Mohammad Ghassemi-Broumand

**Affiliations:** ^1^Department of Optometry, School of Rehabilitation, Shahid Beheshti University of Medical Sciences, Tehran, Iran; ^2^Proteomics Research Center, Department of Biostatistics, School of Allied Medical Sciences, Shahid Beheshti University of Medical Sciences, Tehran, Iran

Dear Editor,

The first region of the visible light spectrum is called blue light. Blue light is beneficial to humans in color vision, night vision, and circadian rhythms.^[1,2]^ However, this type of light raises concerns as it carries high energy and can cause ocular damages, such as photic retinopathy. In addition to the sun, there are several artificial sources of blue light emission, such as light-emitting diodes (LEDs), light bulbs, and fluorescent light tubes. With the increasing use of digital blue-rich LED-backlight displays, such as in mobile devices and tablets, users' eyes are more exposed to blue light.^[2,3]^ Blue light can also induce eyestrain, however, the blue light-blocking lenses may reduce eye fatigue.^[4,5]^

Currently, some lens manufactures claim that their products can alleviate eyestrain and ocular discomfort associated with the use of digital devices.^[5]^ This raises important questions about the efficacy of blue light-control lenses. Therefore, eight blank-white spectacle lenses (four with and four without blue light-blocking property) were collected from different optical companies. A spectrophotometer (Cecil Instrument, UK) was used to measure the blue light transmission. Three ranges of blue light were evaluated: 400–450 nm, 455–500 nm, and 400–500 nm. For the statistical analysis, non-parametric Mann–Whitney test was employed. A *P-value* ≤ 0.05 was considered statistically significant.

**Figure 1:**
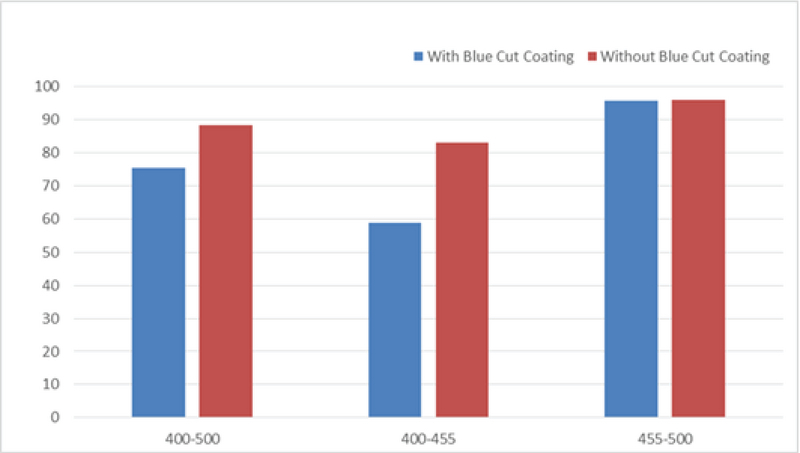
Blue light transmission of lenses with and without blue light-blocking property in different wavelengths.

**Figure 2:**
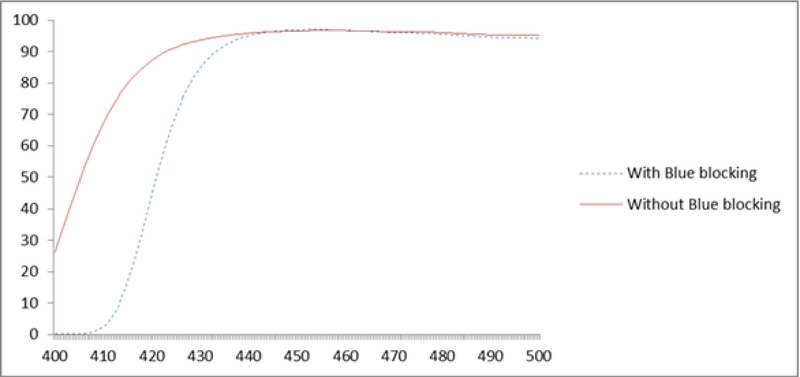
Comparison between spectral transmittance of lenses with and without blue light-blocking property.

The mean transmission of blue light through lenses with and without blue light-blocking coating in the range of 400–455 nm were 58.76 ± 3.01% and 83.10 ± 1.71%, respectively. The differences were statistically significant (*P =* 0.02). The harmful portion of blue light is accumulated in this range as previous studies have shown that using filters capable of 50% reduction in 430 nm blue light transmission can prevent approximately 80% of photochemical damage to the retina. Notably, there is currently no strict guideline for blue light-blocking coatings.^[2]^

The mean transmission of blue light through lenses with and without blue light-blocking coating in the range of 455–500 nm were 95.58 ± 0.46% and 96.00 ± 0.57%, respectively. The differences were not statistically significant (*P* = 0.39). Higher wavelengths, that is, 455–500 nm, are considered useful light for color vision and circadian rhythm.

The mean transmission of blue light through lenses with and without blue light-blocking coating in the range of 400–500 nm were 75.33 ± 1.51% and 88.40 ± 1.63%, respectively. The differences were statistically significant (*P* = 0.02). The lenses with blue light-blocking property could reduce the blue light transmission by approximately 25% in the wavelength range of 400–500 nm. Thus, the filtering value was twice the amount in the lenses without blue light-blocking property (Figures 1 and 2).

Finally, the spectacle lenses with blue light-blocking property could effectively attenuate hazardous lights. It is recommended to use the spectacle lenses that are equipped with blue light-blocking coating to reduce the risk of ocular diseases attributed to hazardous blue light.

##  Conflicts of Interest

There are no conflicts of interest.
